# Supplementary figures and images for: Fat tails and the need to disclose distribution parameters of qEEG databases
Source: PLoS One. 2024 Jan 5;19(1):e0295411. doi: 10.1371/journal.pone.0295411 (PMC10769036; doi:10.1371/journal.pone.0295411)

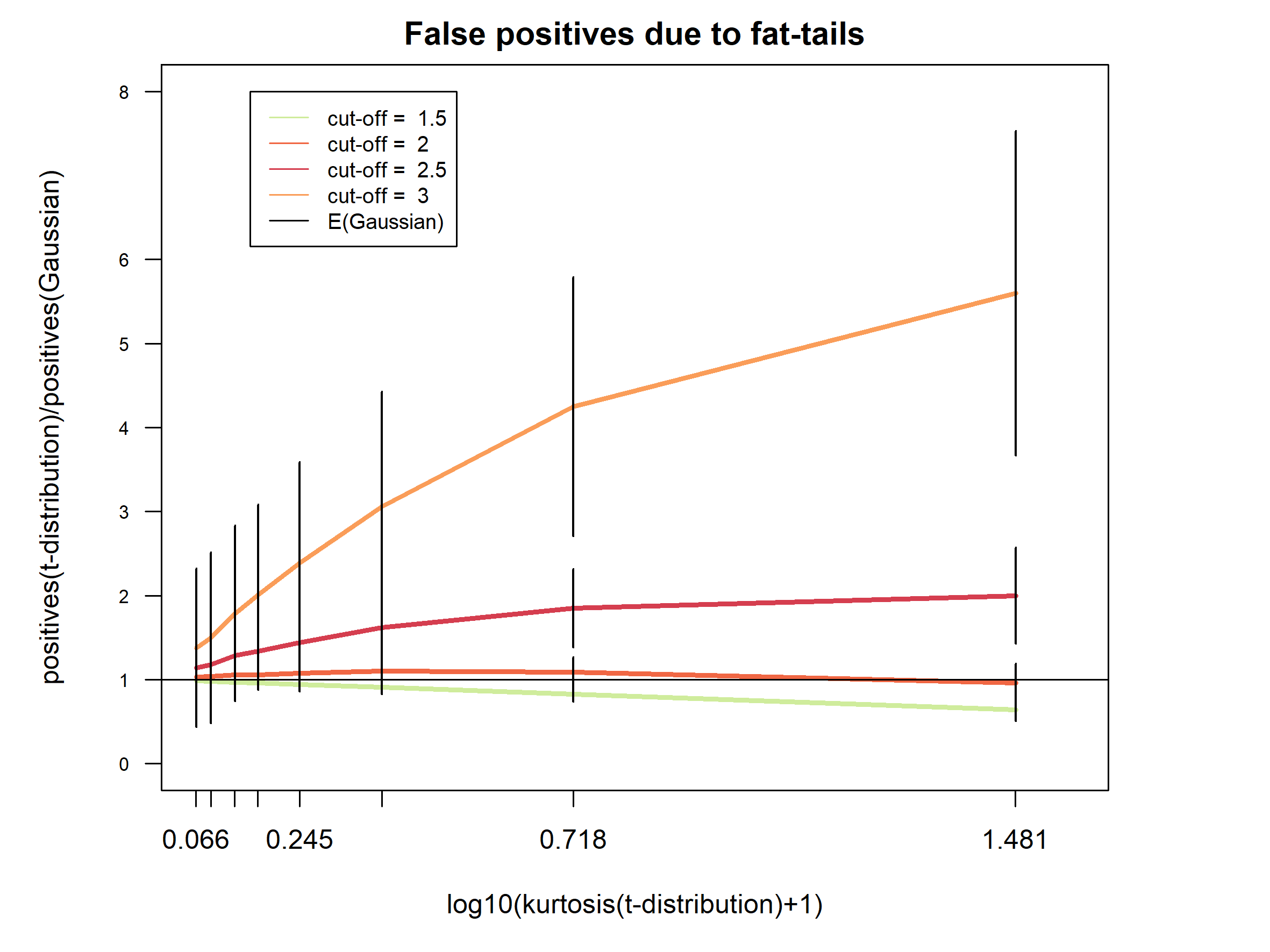

Supplement: S1 Fig — The lower the degrees of freedom of a t-distribution, the more fat-tailed it is. In the S1 Fig the ratio of false-positives(t-distribution)/false-positives(Gaussian) is displayed as a function of the ratio kurtosis(t-distribution)/kurtosis(Gaussian). The larger this last ratio, the more fat-tailed is the distribution. (TIF) [file pone.0295411.s001.tif]

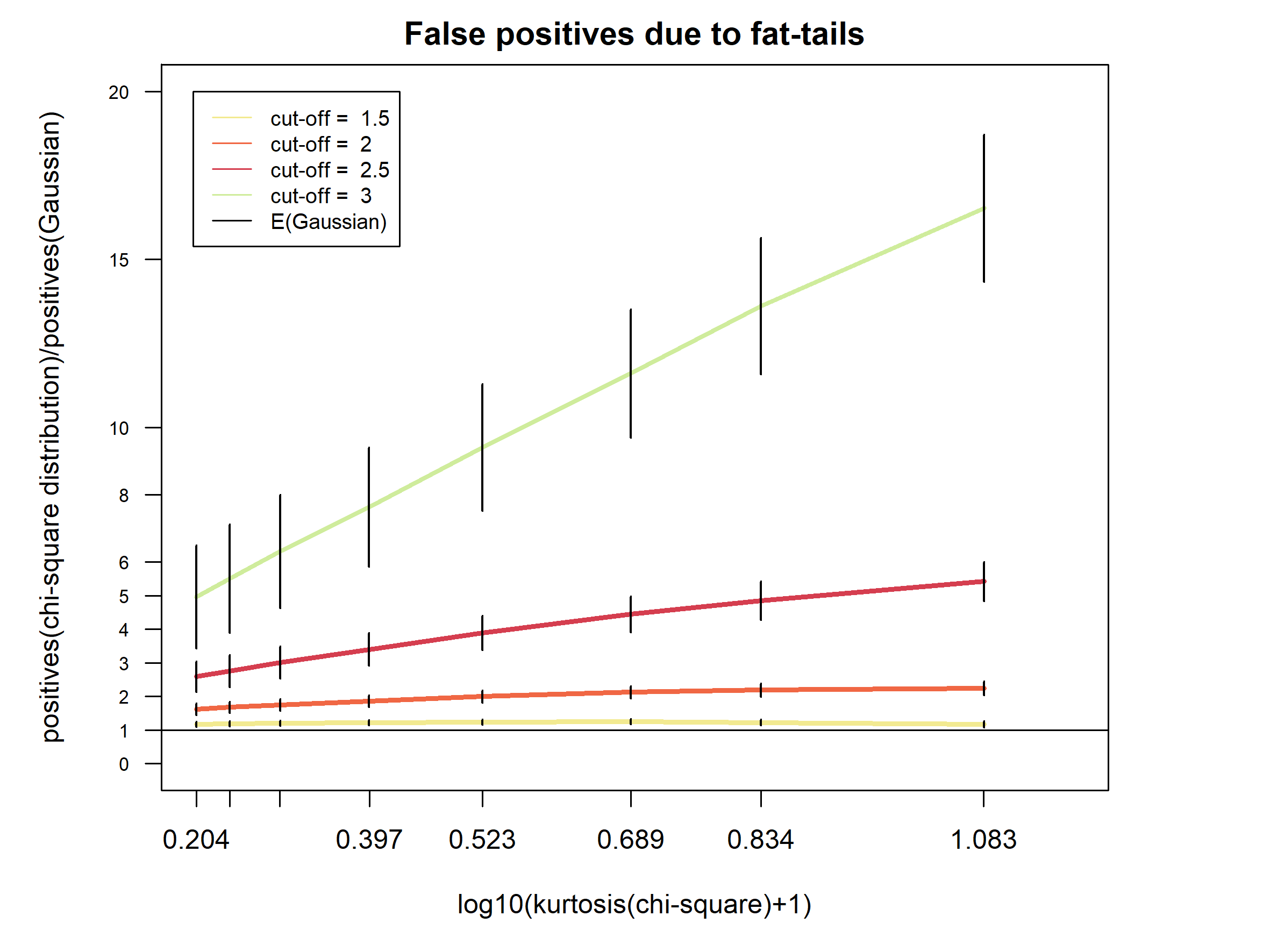

Supplement: S2 Fig — The lower the degrees of freedom of a chi2-distribution, the more fat-tailed it is (Fleishman, 1978, p. 528). In the S2 Fig the ratio of false-positives(chi2-distribution)/false-positives(Gaussian) is displayed as a function of the ratio kurtosis(chi2-distribution)/kurtosis(Gaussian). The larger this last ratio, the more fat-tailed is the distribution. (TIF) [file pone.0295411.s002.tif]

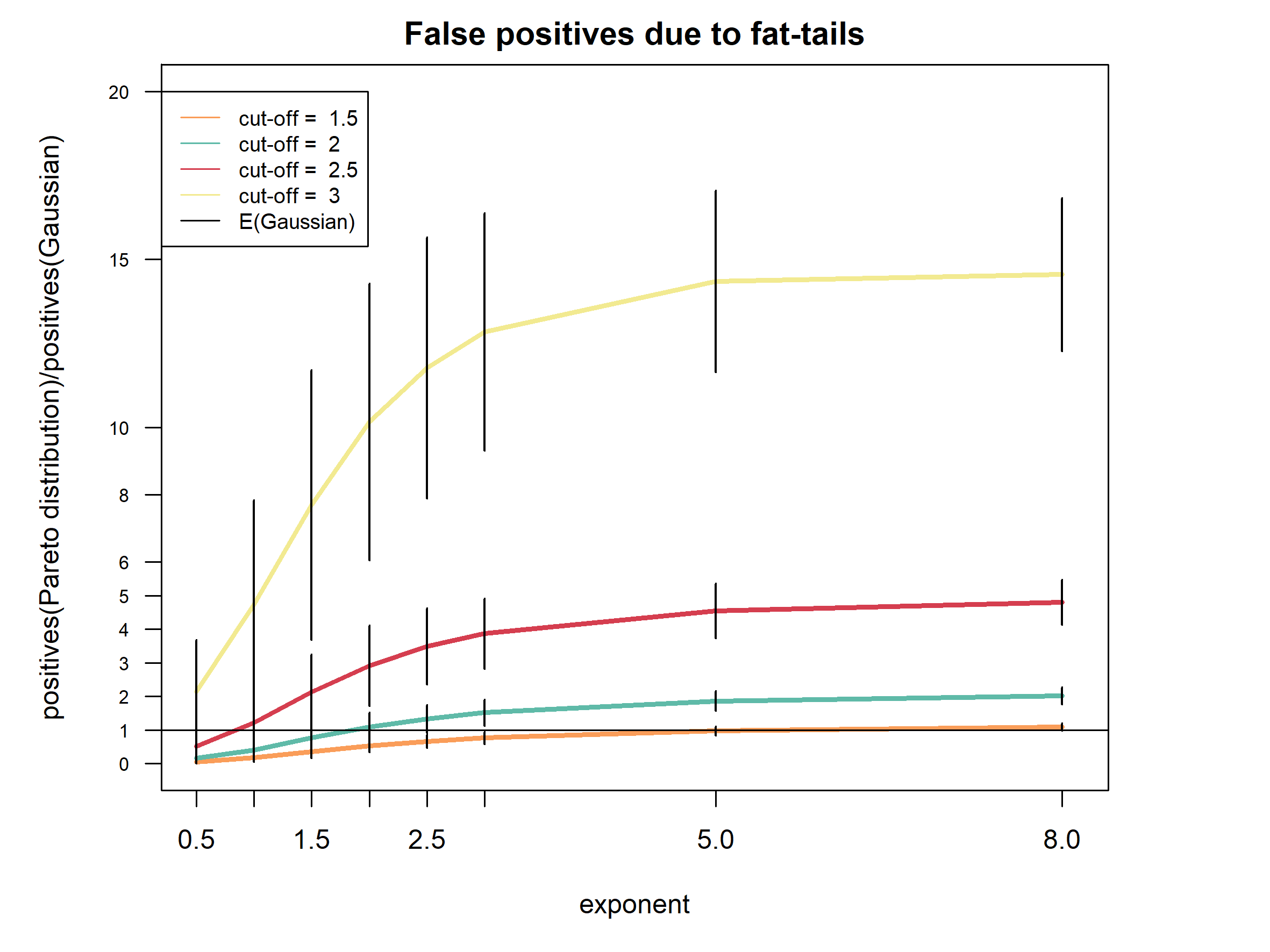

Supplement: S3 Fig — The lower the exponent of a Pareto distribution, the more fat-tailed it is. In the S3 Fig the ratio of false-positives(Pareto distribution)/false-positives(Gaussian) is displayed as a function of the exponent of the distribution. The difference in the x-axis to the other plots is due to the fact that the mean, variance, and other moments are finite only if the shape parameter a is sufficiently large. The larger this last ratio, the more fat-tailed is the distribution. (TIF) [file pone.0295411.s003.tif]
