# Supplementary material for: Fat tails and the need to disclose distribution parameters of qEEG databases
Source: PLoS One. 2024 Jan 5;19(1):e0295411. doi: 10.1371/journal.pone.0295411 (PMC10769036; doi:10.1371/journal.pone.0295411)
Supplement: Attachment — Submitted filename: response_to_reviewers.docx [file pone.0295411.s005.docx]

Additional Editor Comments:
In addition to responding to the reviewer's comments, the author must more clearly and more specific discuss clinical consequence of the inflated abnormal qEEG, related to excess use of unnecessary services. any quantitative discussion on the extra costs?

We are glad to expand on the point of extra costs generated by an excess of false-positive diagnose as follows.

“An inflation of the number of false positive diagnoses of abnormal QEEG has both material and immaterial costs for its users. Material costs can be substantial, for neurofeedback treatment typically takes several sessions to be completed (see tables in Marzbani et al. 2016 for a summary of treat duration), requires the utilization of specialized equipment and time of an expert neurofeedback trainer. In a recent study, Kalokairinou and colleagues (2022) found out that about 73% of users paid for neurofeedback out-of-pocket. As observed by Kalokairinou and colleagues (2022), about 80% of neurofeedback users use it for indications not adequately supported by scientific evidence although more than half (57.6%) considered neurofeedback to be a scientifically well-established therapy. This disconnection between scientific evidence and experiences of users can be considered part of the immaterial costs of neurofeedback. These immaterial costs may be even more substantial for the individuals with a false diagnose of “abnormal” EEG. These persons are confronted with concerns regarding their health and cognitive capabilities. False positive diagnosis is known to induce psychosocial harm in affected individuals (Wakefield, 2010, 2015). Although not as grave as a diagnostic of psychiatric disorder, some form of psychosocial harm can be expected from false-positive EEG diagnosis as well.”

We added this passage to the revised version of the ms.

On the other hand, the fat tail reflect real clinical abnormality? How these fat or non-fat tail (the tail itself) related to clinical conditions. This should be discussed more.

This is an important question. We added he following text passage to the ms. “As we showed in Simulation 2, one consequence of a skewed distribution of EEG is the volatility of more extreme observations, which is much higher than the test-retest reliability would predict, so that typical individuals also produce extreme EEG with a relatively large probability. However, beyond the problems of a psychometric approach to the quantification of EEG we pointed out in the present manuscript, it is still a legitimate question, whether the fat tailedness anyhow reflects real clinical abnormality and how they are related to clinical conditions. Present evidence seems to reveal no clear association between psychiatric disorders and alterations in EEG frequency bands (Nielsen & Thiagarajan, 2019) beyond a trend for elevated power in lower frequency bands and reduced power in higher frequency bands in ADHD, schizophrenia and OCD. The meta-analysis of 184 EEG studies by (Nielsen & Thiagarajan, 2019) compared several psychiatric populations and controls and only revealed small effects and low correlations between EEG and symptom severity. In practical terms, these results support the view that the usefulness of EEG as a marker to evaluate the effect of interventions is in the best case very limited. It is possible that a more detailed analysis of the spatiotemporal features of EEG with the help of deep-learning or other sophisticated mathematical modelling techniques will disclose patterns of abnormal EEG with a higher degree of specificity than traditional neurometry is able to. This is however not yet reality but just speculation. Anyways, understanding tail properties of distributions is usually laborious and mathematically not trivial, requiring a considerable quantity of data which probably cannot be achieved with a few minutes of EEG recording (or less!) as frequently argued in the neurometry literature.”

**Comments to the Author**

5. Review Comments to the Author
Reviewer #1: In their manuscript entitled “Fat tails and the need to disclose distribution parameters of qEEG databases”, the authors use simulation to show that if the normal distribution assumption is violated for the neurometry parameters, the current analyses of qEEG data could be problematic. The authors also make some recommendations for practice in this area.
Although the results are not surprising as we already know that treating non-normal distribution as normally distributed will have undesirable consequences, overall, this is a clearly written report with clinic significance. The authors only simulate log-normal data. It might be more convincing to simulate more data from other (fat-tail) distributions to show the serious limitations when current analyses with normal distribution assumptions are used for qEEG data.

In the revised version of the ms., supplementary materials with the results of simulations using other fat-tailed distributions (t, Chi-square and Pareto distributions with few degrees-of-freedom) are also presented. In all cases, fat-tails led to an inflation in the number of false-positive decisions.

We are thankful for the suggestion to present the results of other distributions, because it led us to find a mistake in the script employed to generate Figure 2. The mistake was corrected and the results presented in the corrected version of Figure 2 kept every aspect of the original interpretation, namely, that a strong inflation in the number of false-positive diagnostic decisions results from the tail properties of the distribution.
